# 6-Benzyl-2-methyl-1,3-bis­(penta­fluoro­phen­yl)-1,3,6,2-triaza­alumocane

**DOI:** 10.1107/S1600536812042560

**Published:** 2012-10-20

**Authors:** Marina M. Kireenko, Kirill V. Zaitsev, Andrei V. Churakov, Galina S. Zaitseva, Sergey S. Karlov

**Affiliations:** a Department of Chemistry, M.V. Lomonosov Moscow State University, Leninskie Gory 1/3, Moscow 119991, Russian Federation; b Institute of General and Inorganic Chemistry, Russian Academy of Sciences, Leninskii prosp. 31, Moscow 119991, Russian Federation

## Abstract

In the title compound, [Al(CH_3_)(C_23_H_15_F_10_N_3_)], the Al^III^ atom is coordinated in a distorted tetra­hedral geometry by three N atoms from the tridentate amine and by one C atom of the methyl substituent. Further, there is a short intra­molecular Al⋯F contact [2.5717 (11) Å], leading to an overall distorted trigonal–bipyramidal coordination environment around Al^III^.

## Related literature

For general background to the chemistry affording the tridentate ligand *N*-benzyl-*N*′-(penta­fluoro­phen­yl)-*N*-{2-[(penta­fluoro­phen­yl)amino]­eth­yl}ethane-1,2-diamine, see: Lermontova *et al.* (2009 ▶). Complexes of germanium and tin based on that and the related ligands and their X-ray structures have been described by Huang *et al.* (2011 ▶, 2012 ▶). For related structures having short Al⋯F—C contacts, see: Smith *et al.* (2010 ▶); Jansen & Mokros (1992 ▶). For a description of the Cambridge Structural Database, see: Allen (2002 ▶).
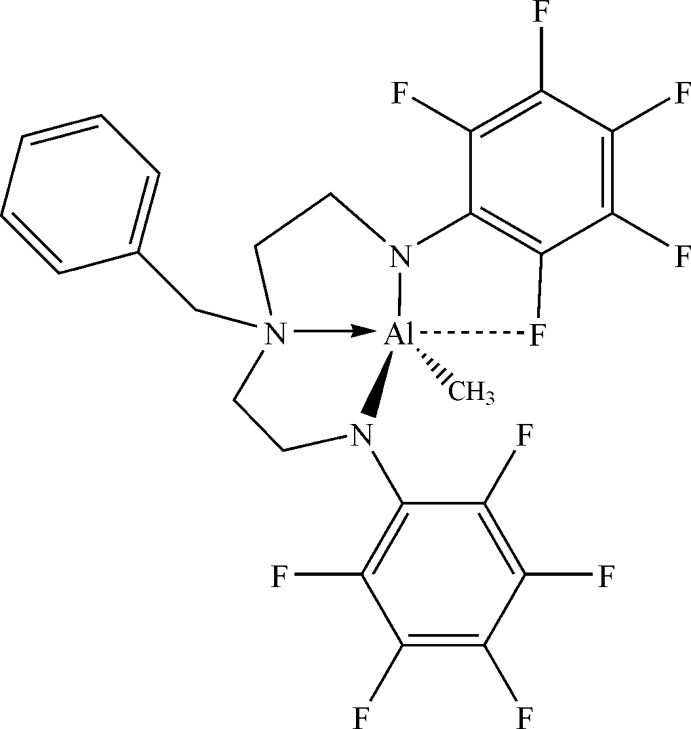


## Experimental

### 

#### Crystal data

[Al(CH_3_)(C_23_H_15_F_10_N_3_)]
*M* *_r_* = 565.39
Monoclinic, 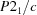

*a* = 7.9530 (14) Å
*b* = 33.577 (6) Å
*c* = 8.7247 (15) Å
β = 100.809 (2)°
*V* = 2288.5 (7) Å^3^
*Z* = 4
Mo *K*α radiation
μ = 0.19 mm^−1^
*T* = 173 K
0.20 × 0.20 × 0.05 mm

#### Data collection

Bruker SMART APEXII diffractometer
Absorption correction: multi-scan (*SADABS*; Bruker, 2008 ▶) *T*_min_ = 0.963, *T*_max_ = 0.991
19211 measured reflections
4972 independent reflections
4317 reflections with *I* > 2σ(*I*)
*R*_int_ = 0.021

#### Refinement

*R*[*F* ^2^ > 2σ(*F* ^2^)] = 0.036
*wR*(*F* ^2^) = 0.094
*S* = 1.02
4972 reflections
415 parameters
All H-atom parameters refined
Δρ_max_ = 0.36 e Å^−3^
Δρ_min_ = −0.26 e Å^−3^

### 

Data collection: *APEX2* (Bruker, 2008 ▶); cell refinement: *SAINT* (Bruker, 2008 ▶); data reduction: *SAINT*; program(s) used to solve structure: *SHELXTL* (Sheldrick, 2008 ▶); program(s) used to refine structure: *SHELXTL*; molecular graphics: *SHELXTL*; software used to prepare material for publication: *SHELXTL*.

## Supplementary Material

Crystal structure: contains datablock(s) global. DOI: 10.1107/S1600536812042560/is5205sup1.cif

Additional supplementary materials:  crystallographic information; 3D view; checkCIF report

## Figures and Tables

**Fig. 1.**
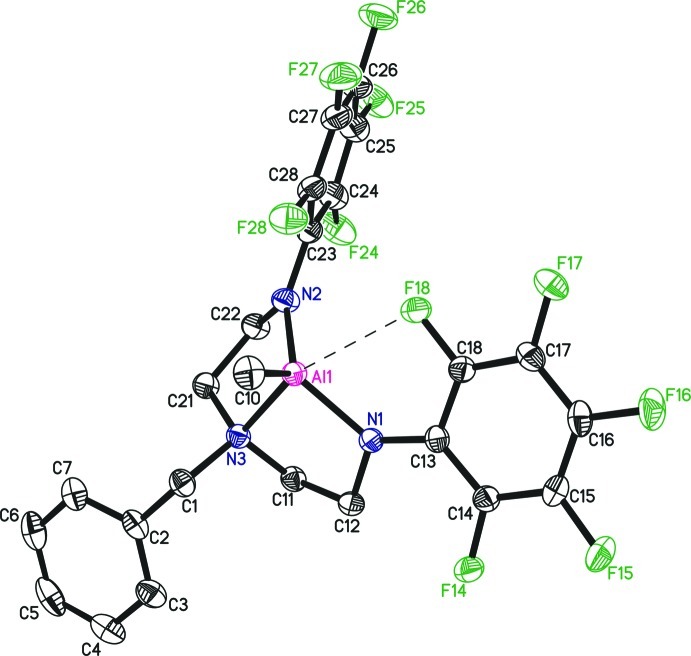
The molecular structure of the title compound, showing the numbering scheme adopted. Displacement ellipsoids are shown at the 50% probability level. The short intramolecular contact Al···F—C is drawn by dashed line. Hydrogen atoms are omitted for clarity.

## supplementary crystallographic information

### Comment

As a part of our investigation on chemistry of germanium, tin and aluminium complexes based on tridentate ligands (Lermontova *et al.*, 2009; Huang *et al.*, 2011, 2012) we obtained and studied the structure of title compound, [Al[C_6_H_5_CH_2_N(CH_2_CH_2_NHC_6_F_5_)_2_](CH_3_)].

The aluminium centre and aromatic F18 atom form a short intramolecular contact [2.5717 (11) Å]. Of interest, F18—C18 bond length [1.359 (2) Å] is the longest among ten F—C distances in the structure. Analysis of Cambridge Structural Database (ver. 5.33, August 2012; Allen, 2002) shows that similar but somewhat longer Al···F—C contacts are observed in the structures EKIBIK (2.690 Å, Smith *et al.*, 2010) and YADTEC (2.719 Å, Jansen & Mokros, 1992).

The Al1 atom has trigonal bipyramid coordination environment. Three equatorial positions are occupied by methyl group C10 and trigonal nitrogen atoms N1 and N2. The angles between C10, N1 and N2 atoms range within 112.86 (7)–124.64 (7)°. The aluminium atom is only slightly displaced [0.1937 (9) Å] from the plane of these ligands towards the coordinated tetrahedral donor atom N3. The N3 and F18 atoms lie in the apical positions with an N3—Al1—F18 angle equal to 152.24 (5)°. As expected, the dative N3→Al1 bond length [2.0104 (13) Å] is significantly longer than equatorial nitrogen-aluminium distances [1.8575 (14) and 1.8697 (14) Å].

### Experimental

A solution of 0.26 g (0.50 mmol) of BnN(CH_2_CH_2_NHC_6_F_5_)_2_ (Lermontova *et al.*, 2009) in toluene (10 ml) was added dropwise to a solution of 0.25 ml (0.50 mmol) of Me_3_Al in toluene (10 ml) under stirring at 243 K. The reaction mixture was allowed to warm to room temperature and stirred overnight. The volatiles were removed under vacuum. The residue was washed by ether (3×5 ml) and dried to give 0.24 g (86%) of BnN(CH_2_CH_2_NHC_6_F_5_)_2_AlMe as a white solid.

^1^H NMR (CDCl_3_): δ -0.56 (br s, 3H, AlMe); 2.88–2.97, 3.00–3.09, 3.57–3.66, 3.67–3.76 (4 m, 8H, NCH_2_); 4.03 (s, 2H, NCH_2_Ph); 7.29–7.34, 7.42–7.47 (2 m, 5H, Ph). ^13^C NMR (CDCl_3_): δ 44.29, 49.94 (4NCH_2_); 56.01 (NCH_2_Ph); 128.77, 129.00, 131.39, 131.55 (Ph). ^1^H NMR (C_6_D_6_): δ -0.31 (p, *J*_F—H_ = 2.4 Hz, 3H, AlMe); 1.95–2.04, 2.10–2.17, 3.08–3.20 (3 m, 8H, NCH_2_); 3.34 (s, 2H, NCH_2_Ph); 6.53–6.57, 7.00–7.06, 7.07–7.10 (3 m, 5H, Ph). ^13^C NMR (C_6_D_6_): δ 49.48, 53.34 (4NCH_2_); 55.61 (NCH_2_Ph); 128.54, 129.24, 129.31, 131.72 (Ph). EI m/*z* 564 (*M*, 5%); 432 (M—Bn, 3%); 91 (Bn, 100%); 42 (AlMe, 78%). Anal. Calcd. for C_24_H_18_AlN_3_F_10_: C 50.98, H 3.21, N 7.43. Found: C 50.68, H 3.36, N 7.39.

The crystals were obtained from the concentrated toluene solution under storing at 255 K for several days.

### Refinement

All H atoms were located in a difference Fourier map and refined isotropically. [Refined C—H distances: 0.90 (2)–0.97 (3) Å for aromatic CH, 0.931 (19)–1.004 (19) Å for CH_2_ and 0.92 (3)–0.98 (2) Å for CH_3_.]

### Crystal data

**Table:**

[Al(CH_3_)(C_23_H_15_F_10_N_3_)]	*F*(000) = 1144
*M**_r_* = 565.39	*D*_x_ = 1.641 Mg m^−^^3^
Monoclinic, *P*2_1_/*c*	Mo *K*α radiation, λ = 0.71073 Å
Hall symbol: -P 2ybc	Cell parameters from 8521 reflections
*a* = 7.9530 (14) Å	θ = 2.5–28.1°
*b* = 33.577 (6) Å	µ = 0.19 mm^−^^1^
*c* = 8.7247 (15) Å	*T* = 173 K
β = 100.809 (2)°	Plate, colourless
*V* = 2288.5 (7) Å^3^	0.20 × 0.20 × 0.05 mm
*Z* = 4	

### Data collection

**Table:**

Bruker SMART APEXII diffractometer	4972 independent reflections
Radiation source: fine-focus sealed tube	4317 reflections with *I* > 2σ(*I*)
Graphite monochromator	*R*_int_ = 0.021
ω scans	θ_max_ = 27.0°, θ_min_ = 2.4°
Absorption correction: multi-scan (*SADABS*; Bruker, 2008)	*h* = −10→10
*T*_min_ = 0.963, *T*_max_ = 0.991	*k* = −42→42
19211 measured reflections	*l* = −11→11

### Refinement

**Table:**

Refinement on *F*^2^	Primary atom site location: structure-invariant direct methods
Least-squares matrix: full	Secondary atom site location: difference Fourier map
*R*[*F*^2^ > 2σ(*F*^2^)] = 0.036	Hydrogen site location: difference Fourier map
*wR*(*F*^2^) = 0.094	All H-atom parameters refined
*S* = 1.02	*w* = 1/[σ^2^(*F*_o_^2^) + (0.0439*P*)^2^ + 1.3304*P*] where *P* = (*F*_o_^2^ + 2*F*_c_^2^)/3
4972 reflections	(Δ/σ)_max_ < 0.001
415 parameters	Δρ_max_ = 0.36 e Å^−^^3^
0 restraints	Δρ_min_ = −0.26 e Å^−^^3^

### Special details

**Table:**

Geometry. All e.s.d.'s (except the e.s.d. in the dihedral angle between two l.s. planes) are estimated using the full covariance matrix. The cell e.s.d.'s are taken into account individually in the estimation of e.s.d.'s in distances, angles and torsion angles; correlations between e.s.d.'s in cell parameters are only used when they are defined by crystal symmetry. An approximate (isotropic) treatment of cell e.s.d.'s is used for estimating e.s.d.'s involving l.s. planes.
Refinement. Refinement of *F*^2^ against ALL reflections. The weighted *R*-factor *wR* and goodness of fit *S* are based on *F*^2^, conventional *R*-factors *R* are based on *F*, with *F* set to zero for negative *F*^2^. The threshold expression of *F*^2^ > σ(*F*^2^) is used only for calculating *R*-factors(gt) *etc*. and is not relevant to the choice of reflections for refinement. *R*-factors based on *F*^2^ are statistically about twice as large as those based on *F*, and *R*- factors based on ALL data will be even larger.

### Fractional atomic coordinates and isotropic or equivalent isotropic displacement parameters (Å^2^)

**Table:**

	*x*	*y*	*z*	*U*_iso_*/*U*_eq_	
Al1	0.55982 (6)	0.117782 (13)	0.49859 (5)	0.02089 (11)	
N1	0.60780 (16)	0.16461 (4)	0.39907 (14)	0.0223 (3)	
N2	0.68539 (17)	0.07207 (4)	0.47836 (15)	0.0254 (3)	
N3	0.74288 (16)	0.13396 (4)	0.67940 (14)	0.0214 (3)	
C1	0.6675 (2)	0.15076 (5)	0.81197 (18)	0.0247 (3)	
C2	0.7938 (2)	0.16032 (5)	0.95983 (18)	0.0267 (3)	
C3	0.8602 (2)	0.19843 (5)	0.9886 (2)	0.0308 (4)	
C4	0.9723 (2)	0.20708 (6)	1.1272 (2)	0.0375 (4)	
C5	1.0209 (2)	0.17743 (7)	1.2368 (2)	0.0408 (5)	
C6	0.9549 (3)	0.13976 (7)	1.2102 (2)	0.0407 (5)	
C7	0.8404 (2)	0.13109 (6)	1.0735 (2)	0.0340 (4)	
C10	0.3337 (2)	0.11830 (6)	0.5555 (2)	0.0304 (4)	
C11	0.84769 (19)	0.16404 (5)	0.61234 (18)	0.0236 (3)	
C12	0.7313 (2)	0.19083 (5)	0.49746 (18)	0.0232 (3)	
C13	0.51390 (18)	0.17824 (5)	0.26115 (17)	0.0212 (3)	
C14	0.4895 (2)	0.21746 (5)	0.20674 (18)	0.0235 (3)	
C15	0.3887 (2)	0.22716 (5)	0.06498 (19)	0.0261 (3)	
C16	0.3087 (2)	0.19796 (5)	−0.03367 (18)	0.0279 (3)	
C17	0.3261 (2)	0.15883 (5)	0.01513 (18)	0.0257 (3)	
C18	0.4249 (2)	0.15007 (5)	0.15781 (18)	0.0235 (3)	
C21	0.8424 (2)	0.09669 (5)	0.72549 (19)	0.0258 (3)	
C22	0.8566 (2)	0.07283 (5)	0.58014 (19)	0.0270 (3)	
C23	0.6498 (2)	0.04053 (5)	0.37471 (18)	0.0274 (3)	
C24	0.7699 (2)	0.01889 (5)	0.3099 (2)	0.0338 (4)	
C25	0.7253 (3)	−0.01322 (6)	0.2104 (2)	0.0405 (5)	
C26	0.5577 (3)	−0.02420 (5)	0.1651 (2)	0.0408 (5)	
C27	0.4342 (3)	−0.00290 (5)	0.2209 (2)	0.0371 (4)	
C28	0.4803 (2)	0.02780 (5)	0.3244 (2)	0.0304 (4)	
F14	0.56225 (13)	0.24841 (3)	0.29513 (11)	0.0310 (2)	
F15	0.36334 (13)	0.26570 (3)	0.02492 (12)	0.0354 (2)	
F16	0.21053 (13)	0.20771 (3)	−0.17192 (11)	0.0392 (3)	
F17	0.24581 (13)	0.12973 (3)	−0.07535 (12)	0.0371 (2)	
F18	0.43879 (13)	0.11143 (3)	0.20488 (11)	0.0309 (2)	
F24	0.93701 (14)	0.02933 (4)	0.34006 (13)	0.0452 (3)	
F25	0.84828 (18)	−0.03270 (4)	0.15369 (15)	0.0569 (4)	
F26	0.51315 (19)	−0.05520 (3)	0.06690 (14)	0.0553 (4)	
F27	0.26773 (16)	−0.01208 (3)	0.17435 (14)	0.0492 (3)	
F28	0.35477 (13)	0.04643 (3)	0.37978 (13)	0.0382 (3)	
H112	0.917 (2)	0.1790 (5)	0.6947 (19)	0.018 (4)*	
H122	0.803 (2)	0.2058 (5)	0.436 (2)	0.020 (4)*	
H121	0.675 (2)	0.2111 (5)	0.554 (2)	0.020 (4)*	
H222	0.894 (2)	0.0457 (6)	0.615 (2)	0.027 (5)*	
H3	0.833 (2)	0.2184 (5)	0.912 (2)	0.025 (4)*	
H12	0.605 (2)	0.1750 (6)	0.774 (2)	0.030 (5)*	
H111	0.921 (2)	0.1507 (5)	0.558 (2)	0.024 (4)*	
H212	0.778 (2)	0.0818 (5)	0.790 (2)	0.028 (5)*	
H221	0.950 (2)	0.0841 (6)	0.530 (2)	0.028 (5)*	
H11	0.585 (2)	0.1307 (6)	0.834 (2)	0.031 (5)*	
H211	0.955 (2)	0.1031 (6)	0.787 (2)	0.029 (5)*	
H7	0.792 (2)	0.1051 (6)	1.055 (2)	0.031 (5)*	
H103	0.310 (3)	0.1447 (7)	0.596 (3)	0.050 (6)*	
H4	1.012 (3)	0.2322 (7)	1.140 (2)	0.036 (5)*	
H5	1.097 (3)	0.1834 (6)	1.326 (3)	0.045 (6)*	
H102	0.322 (3)	0.0973 (7)	0.630 (3)	0.048 (6)*	
H6	0.982 (3)	0.1182 (7)	1.285 (3)	0.060 (7)*	
H101	0.255 (3)	0.1136 (8)	0.465 (3)	0.064 (7)*	

### Atomic displacement parameters (Å^2^)

**Table:**

	*U*^11^	*U*^22^	*U*^33^	*U*^12^	*U*^13^	*U*^23^
Al1	0.0208 (2)	0.0198 (2)	0.0209 (2)	0.00011 (17)	0.00084 (17)	0.00025 (17)
N1	0.0248 (6)	0.0209 (6)	0.0193 (6)	−0.0020 (5)	−0.0004 (5)	0.0001 (5)
N2	0.0265 (7)	0.0217 (6)	0.0254 (7)	0.0034 (5)	−0.0016 (5)	−0.0024 (5)
N3	0.0222 (6)	0.0213 (6)	0.0201 (6)	0.0019 (5)	0.0022 (5)	−0.0006 (5)
C1	0.0233 (7)	0.0286 (8)	0.0216 (7)	0.0019 (6)	0.0028 (6)	−0.0012 (6)
C2	0.0231 (7)	0.0362 (9)	0.0208 (7)	0.0034 (6)	0.0042 (6)	−0.0041 (6)
C3	0.0287 (8)	0.0338 (9)	0.0293 (8)	0.0055 (7)	0.0038 (7)	−0.0060 (7)
C4	0.0307 (9)	0.0450 (11)	0.0368 (10)	−0.0016 (8)	0.0066 (7)	−0.0164 (8)
C5	0.0278 (9)	0.0717 (14)	0.0219 (8)	0.0035 (9)	0.0016 (7)	−0.0107 (9)
C6	0.0403 (10)	0.0600 (13)	0.0212 (8)	0.0057 (9)	0.0042 (7)	0.0045 (8)
C7	0.0361 (9)	0.0418 (10)	0.0242 (8)	0.0004 (8)	0.0059 (7)	0.0013 (7)
C10	0.0238 (8)	0.0363 (10)	0.0303 (9)	0.0002 (7)	0.0031 (7)	0.0010 (8)
C11	0.0217 (7)	0.0258 (8)	0.0223 (7)	−0.0029 (6)	0.0020 (6)	−0.0018 (6)
C12	0.0238 (7)	0.0222 (7)	0.0230 (7)	−0.0033 (6)	0.0024 (6)	−0.0022 (6)
C13	0.0200 (7)	0.0241 (7)	0.0201 (7)	0.0002 (6)	0.0051 (5)	0.0007 (6)
C14	0.0241 (7)	0.0227 (7)	0.0237 (7)	−0.0022 (6)	0.0049 (6)	−0.0006 (6)
C15	0.0261 (8)	0.0254 (8)	0.0273 (8)	0.0012 (6)	0.0065 (6)	0.0075 (6)
C16	0.0229 (7)	0.0391 (9)	0.0205 (7)	0.0007 (7)	0.0007 (6)	0.0063 (7)
C17	0.0232 (7)	0.0313 (8)	0.0223 (7)	−0.0039 (6)	0.0034 (6)	−0.0045 (6)
C18	0.0250 (7)	0.0221 (7)	0.0236 (7)	0.0008 (6)	0.0054 (6)	−0.0003 (6)
C21	0.0262 (8)	0.0249 (8)	0.0237 (8)	0.0044 (6)	−0.0018 (6)	0.0008 (6)
C22	0.0256 (8)	0.0256 (8)	0.0274 (8)	0.0059 (6)	−0.0017 (6)	−0.0017 (6)
C23	0.0370 (9)	0.0188 (7)	0.0238 (8)	0.0026 (6)	−0.0005 (6)	0.0020 (6)
C24	0.0374 (9)	0.0299 (9)	0.0310 (9)	0.0079 (7)	−0.0020 (7)	−0.0021 (7)
C25	0.0567 (12)	0.0293 (9)	0.0326 (9)	0.0163 (8)	0.0013 (8)	−0.0025 (7)
C26	0.0650 (13)	0.0200 (8)	0.0313 (9)	0.0015 (8)	−0.0064 (9)	−0.0029 (7)
C27	0.0492 (11)	0.0227 (8)	0.0343 (9)	−0.0062 (8)	−0.0050 (8)	0.0036 (7)
C28	0.0389 (9)	0.0200 (8)	0.0302 (8)	−0.0011 (7)	0.0008 (7)	0.0029 (6)
F14	0.0387 (5)	0.0212 (5)	0.0299 (5)	−0.0042 (4)	−0.0014 (4)	0.0004 (4)
F15	0.0387 (6)	0.0295 (5)	0.0362 (5)	0.0026 (4)	0.0024 (4)	0.0130 (4)
F16	0.0365 (6)	0.0502 (7)	0.0257 (5)	−0.0025 (5)	−0.0074 (4)	0.0107 (5)
F17	0.0392 (6)	0.0393 (6)	0.0286 (5)	−0.0072 (4)	−0.0045 (4)	−0.0082 (4)
F18	0.0384 (5)	0.0214 (5)	0.0300 (5)	−0.0022 (4)	−0.0009 (4)	−0.0010 (4)
F24	0.0363 (6)	0.0525 (7)	0.0453 (6)	0.0106 (5)	0.0041 (5)	−0.0165 (5)
F25	0.0693 (8)	0.0504 (7)	0.0465 (7)	0.0275 (6)	−0.0010 (6)	−0.0186 (6)
F26	0.0880 (10)	0.0271 (6)	0.0431 (7)	0.0011 (6)	−0.0074 (6)	−0.0131 (5)
F27	0.0533 (7)	0.0347 (6)	0.0531 (7)	−0.0174 (5)	−0.0067 (6)	−0.0040 (5)
F28	0.0328 (5)	0.0360 (6)	0.0452 (6)	−0.0056 (4)	0.0054 (5)	−0.0057 (5)

### Geometric parameters (Å, º)

**Table:**

Al1—N2	1.8575 (14)	C11—H111	0.931 (19)
Al1—N1	1.8697 (14)	C12—H122	0.990 (17)
Al1—C10	1.9536 (18)	C12—H121	0.995 (17)
Al1—N3	2.0104 (13)	C13—C14	1.401 (2)
Al1—F18	2.5717 (11)	C13—C18	1.403 (2)
N1—C13	1.3704 (19)	C14—F14	1.3573 (17)
N1—C12	1.4700 (19)	C14—C15	1.381 (2)
N2—C23	1.387 (2)	C15—F15	1.3457 (18)
N2—C22	1.480 (2)	C15—C16	1.379 (2)
N3—C21	1.4947 (19)	C16—F16	1.3486 (18)
N3—C11	1.497 (2)	C16—C17	1.380 (2)
N3—C1	1.5086 (19)	C17—F17	1.3404 (18)
C1—C2	1.514 (2)	C17—C18	1.373 (2)
C1—H12	0.976 (19)	C18—F18	1.3591 (18)
C1—H11	0.99 (2)	C21—C22	1.522 (2)
C2—C3	1.389 (2)	C21—H212	0.972 (19)
C2—C7	1.395 (2)	C21—H211	0.980 (19)
C3—C4	1.392 (2)	C22—H222	0.987 (19)
C3—H3	0.946 (19)	C22—H221	1.004 (19)
C4—C5	1.384 (3)	C23—C24	1.401 (2)
C4—H4	0.90 (2)	C23—C28	1.404 (2)
C5—C6	1.372 (3)	C24—F24	1.352 (2)
C5—H5	0.91 (2)	C24—C25	1.388 (3)
C6—C7	1.390 (3)	C25—F25	1.345 (2)
C6—H6	0.97 (3)	C25—C26	1.368 (3)
C7—H7	0.96 (2)	C26—F26	1.353 (2)
C10—H103	0.98 (2)	C26—C27	1.375 (3)
C10—H102	0.98 (2)	C27—F27	1.346 (2)
C10—H101	0.92 (3)	C27—C28	1.374 (2)
C11—C12	1.524 (2)	C28—F28	1.342 (2)
C11—H112	0.963 (17)		
			
N2—Al1—N1	119.38 (6)	H112—C11—H111	107.5 (14)
N2—Al1—C10	124.64 (7)	N1—C12—C11	106.70 (12)
N1—Al1—C10	112.86 (7)	N1—C12—H122	112.0 (10)
N2—Al1—N3	88.55 (6)	C11—C12—H122	108.6 (10)
N1—Al1—N3	87.50 (6)	N1—C12—H121	112.8 (10)
C10—Al1—N3	111.33 (7)	C11—C12—H121	110.5 (10)
N2—Al1—F18	86.80 (5)	H122—C12—H121	106.2 (14)
N1—Al1—F18	71.20 (4)	N1—C13—C14	129.02 (14)
C10—Al1—F18	93.65 (6)	N1—C13—C18	117.72 (14)
N3—Al1—F18	152.24 (5)	C14—C13—C18	113.25 (13)
C13—N1—C12	120.47 (12)	F14—C14—C15	116.26 (14)
C13—N1—Al1	124.31 (10)	F14—C14—C13	120.67 (13)
C12—N1—Al1	113.87 (9)	C15—C14—C13	123.05 (14)
C23—N2—C22	117.19 (13)	F15—C15—C16	119.51 (14)
C23—N2—Al1	130.18 (11)	F15—C15—C14	119.53 (14)
C22—N2—Al1	112.29 (10)	C16—C15—C14	120.92 (15)
C21—N3—C11	111.39 (12)	F16—C16—C15	120.50 (15)
C21—N3—C1	111.92 (12)	F16—C16—C17	120.99 (15)
C11—N3—C1	112.20 (12)	C15—C16—C17	118.46 (14)
C21—N3—Al1	104.70 (9)	F17—C17—C18	120.41 (15)
C11—N3—Al1	104.48 (9)	F17—C17—C16	120.21 (14)
C1—N3—Al1	111.67 (9)	C18—C17—C16	119.37 (14)
N3—C1—C2	115.91 (12)	F18—C18—C17	118.59 (13)
N3—C1—H12	107.5 (11)	F18—C18—C13	116.52 (13)
C2—C1—H12	109.0 (11)	C17—C18—C13	124.89 (15)
N3—C1—H11	105.8 (11)	N3—C21—C22	109.57 (12)
C2—C1—H11	109.8 (11)	N3—C21—H212	106.1 (11)
H12—C1—H11	108.7 (15)	C22—C21—H212	110.1 (11)
C3—C2—C7	118.60 (15)	N3—C21—H211	110.3 (11)
C3—C2—C1	121.23 (15)	C22—C21—H211	111.8 (11)
C7—C2—C1	120.12 (16)	H212—C21—H211	108.7 (15)
C2—C3—C4	120.60 (18)	N2—C22—C21	107.78 (13)
C2—C3—H3	119.8 (11)	N2—C22—H222	111.0 (10)
C4—C3—H3	119.5 (11)	C21—C22—H222	107.3 (11)
C5—C4—C3	120.01 (19)	N2—C22—H221	114.4 (10)
C5—C4—H4	122.9 (13)	C21—C22—H221	110.1 (11)
C3—C4—H4	117.1 (13)	H222—C22—H221	106.0 (15)
C6—C5—C4	119.88 (17)	N2—C23—C24	126.01 (16)
C6—C5—H5	121.3 (14)	N2—C23—C28	120.22 (15)
C4—C5—H5	118.9 (14)	C24—C23—C28	113.77 (15)
C5—C6—C7	120.41 (19)	F24—C24—C25	116.72 (16)
C5—C6—H6	123.0 (15)	F24—C24—C23	120.44 (15)
C7—C6—H6	116.6 (15)	C25—C24—C23	122.82 (17)
C6—C7—C2	120.45 (19)	F25—C25—C26	119.94 (17)
C6—C7—H7	121.0 (12)	F25—C25—C24	119.32 (19)
C2—C7—H7	118.6 (12)	C26—C25—C24	120.70 (18)
Al1—C10—H103	109.9 (13)	F26—C26—C25	121.03 (19)
Al1—C10—H102	111.6 (13)	F26—C26—C27	120.31 (18)
H103—C10—H102	111.6 (19)	C25—C26—C27	118.66 (16)
Al1—C10—H101	106.8 (16)	F27—C27—C28	119.72 (18)
H103—C10—H101	108 (2)	F27—C27—C26	120.11 (16)
H102—C10—H101	108 (2)	C28—C27—C26	120.17 (18)
N3—C11—C12	109.96 (12)	F28—C28—C27	117.51 (16)
N3—C11—H112	110.2 (10)	F28—C28—C23	118.72 (14)
C12—C11—H112	112.0 (10)	C27—C28—C23	123.77 (17)
N3—C11—H111	108.8 (11)	C18—F18—Al1	102.48 (8)
C12—C11—H111	108.3 (11)		
